# Associations of physical activity with sleep satisfaction, perceived stress, and problematic Internet use in Korean adolescents

**DOI:** 10.1186/1471-2458-14-1143

**Published:** 2014-11-05

**Authors:** Subin Park

**Affiliations:** Department of Psychiatry, Seoul National Hospital 398, Neungdong-ro, Gwangin-gu, Seoul, 143-711 South Korea

**Keywords:** Epidemiology, Mental Health, Exercise, KYRBS, Adolescents

## Abstract

**Background:**

The association of physical activity (PA) with sleep quality, perceived stress, and problematic Internet use was examined in a nationwide sample of Korean adolescents.

**Methods:**

Data from the 2010 Korean Youth Risk Behavior Web-based Survey collected from 73,238 Korean adolescents aged 12–18 years (mean age 15.06 ± 1.75 years) were analyzed. Participants were asked to rate the frequency with which they engaged in moderate and vigorous PA per week. The risk of problematic Internet use was assessed with the Korean Internet Addiction Proneness Scale for Youth-Short Form. Self-report questionnaires were used to assess levels of sleep satisfaction and perceived stress. The associations of PA with sleep satisfaction, perceived stress, and problematic Internet use were assessed with multiple logistic regression analysis. Then, the Sobel test was used to explore the mediation of the relationship between PA and problematic Internet use by sleep satisfaction and stress.

**Results:**

Physically active subjects were more likely to express satisfaction with their sleep (*AOR* = 1.13; 95% *CI* = 1.08, 1.18), less likely to feel stress in their lives (*AOR* = 0.89, 95% *CI* = 0.86, 0.93), and less likely to be a problematic Internet user (*AOR* = 0.78; 95% *CI* = 0.73, 0.82) compared to physically inactive subjects. The Sobel test revealed that the inverse association between PA and problematic Internet use was partially mediated by increased sleep satisfaction or decreased perceived stress (*Z* = −4.315, *p* < 0.001).

**Conclusions:**

The results of the present study indicate a negative association between level of physical activity and risk of problematic Internet use via the mediation of sleep satisfaction and stress in Korean adolescents. Physical activity may be helpful to improve adolescent mental health.

## Background

Adolescence is an important developmental stage during which many habits related to a healthy lifestyle, such as those involving physical activity (PA) and quality of sleep, are established. Participation in PA has been associated with higher self-esteem [1], increased physical and psychological well-being [2–4], and reduced risk of depression and anxiety [5] in adolescence. In contrast, poor sleep has been associated with maladaptive functioning and a number of psychiatric and physical conditions [6].

In South Korea, where more than 90% of the population has access to the Internet, problematic Internet use is becoming a serious social problem, especially among adolescents [7]. Problematic Internet use is characterized by an inability to control use of the Internet, resulting in marked distress and/or functional impairment [8]. Previous studies have found problematic Internet use in about 10–30% of Korean junior or senior high school students [8–11], and this behavior was strongly associated with depression and suicide [8–10, 12–14].

Extensive use of the Internet and computers may be inversely associated with PA because it involves time that might otherwise be spent engaging in PA [15]. In addition to this direct relationship between Internet use and low PA, PA may protect against problematic Internet use via its positive effect on stress and sleep. Problematic Internet use may constitute a coping mechanism by which adolescents temporarily relieve and/or escape from emotional difficulties and stress [16]. However, PA allows for a discharge of hostility and serves as a buffer against stressful events [17]. Adolescents who are more physically active may be less likely to engage in excessive Internet to cope with stress. Moreover, adolescents who stay up late chatting, gaming, or engaging in other Internet activities may experience insufficient or disrupted sleep [18]. A study among Finnish adolescents [19] found that intensive computer use among boys was associated with poor sleeping habits and tiredness while awake. In contrast, PA was positively associated with the restoration of healthy sleep habits and improvement of sleep disturbances [20].

Building on previous research in this area, this study investigated the complex relationships among PA, sleep satisfaction, perceived stress, and problematic Internet use among a large sample of Korean adolescents. Based on the previous reports of positive effect of PA on reduction of stress and sleep disturbance [17, 20], which are associated with problematic internet use [16, 19], it was hypothesized that 1) PA is related to problematic Internet use and 2) this tentative association is mediated by sleep satisfaction and perceived stress.

## Methods

### Subjects

Data from the 2010 Korean Youth Risk Behavior Web-based Survey (KYRBS) was used. The KYRBS is a government-approved statistical survey that has been performed annually by the Ministry of Education, Ministry of Health and Welfare, and Korea Centers for Disease Control and Prevention since 2005 to monitor health-related risk behaviors among Korean adolescents. The 2010 KYRBS was conducted from September 1, 2010, to October 24, 2010, using a stratified multistage cluster sampling design to obtain a nationally representative sample of middle and high school students. Students voluntarily completed the anonymous, self-administered web-based survey during a regular class period. Written informed consent was not obtained from participants because KYRBS did not collect any personal information such as the students’ names, their school, telephone number, home address, or social security number. A total of 74,980 students from 400 middle schools and 400 high schools participated in the survey, representing a response rate of 97.7%. The final sample included 73,238 students (38,391 boys and 34,847 girls, mean age 15.06 ± 1.75 years, range 12–18 years). Additional details about the sampling methodology and survey procedure are available elsewhere [21]. This study was reviewed and approved by the institutional review board of Seoul National University Hospital.

### Measurements

Sociodemographic variables included sex, age, place of residence (name of city), perceived academic performance, perceived family economic status, and parents’ level of education. Places of residence were classified into rural area, small city, and large city. Respondents were asked to self-record their height and weight, and their body mass index (kg/m^2^) was calculated based on these data.

The frequency of moderate-intensity PA was assessed with the following question: “In the last week, on how many days did you engage in 30 minutes or more of physical activity that increased your heart rate or breathing rate (e.g., cycling at a regular pace, carrying light loads, or playing doubles tennis)?”. The frequency of vigorous-intensity PA was assessed with the following question, “In the last week, on how many days did you engage in 20 minutes or more of physical that was so vigorous it left you soaked with perspirationor breathless (e.g., digging, aerobics, heavy lifting, or fast cycling)?”. The response options ranged from 1 (none) to 6 (more than 5 days per week). The two criteria used for classification as physically active were: a) vigorous activity at least 3 days per week, OR b) moderate activity at least 5 days per week. These criteria were based on the criteria of moderate level of PA of International Physical Activity Questionnaire (IPAQ) Scoring Protocol [22].

Problematic Internet use was measured with the Internet Addiction Proneness Scale for Youth–Short Form (KS scale) developed by the Korean National Information Society Agency [8, 13]. The KS scale is a 20-item self-report instrument administered to screen youth who are prone to problematic Internet use. Items are rated on a four-point Likert scale (1 = never, 2 = sometimes, 3 = often, or 4 = nearly always). It consists of six sub-factors: (1) disturbance of adaptive functioning, (2) addictive automatic thought, (3) withdrawal, (4) virtual interpersonal relationship, (5) deviant behavior, and (6) tolerance. The validity and reliability of the KS scale was established for elementary school and junior and senior high school students, separately [13]. In the case of junior and senior high school students, the Cronbach’s alpha score was 0.909 and Internet addiction was defined by a total score higher than 53 or the presence of all of the following: adaptive functioning scores higher than 17; withdrawal scores higher than 11; and tolerance scores higher than 13. Probable Internet addiction was defined by the presence of one of the following: a total score between 48 and 52, adaptive functioning scores higher than 15, withdrawal scores higher than 10, or tolerance scores higher than 12. In this study, both definite and probable Internet addicts were included inthe problematic Internet-use group.

The level of perceived stress was measured with the following question: “How much stress do you usually feel?” The response options were very little (1), a little (2), an average amount (3), a lot (4), and very much (5). On the basis of the responses, the participants were classified into the following two groups for multivariate logistic regression analyses: ≤ average perceived stress (1–3) and (ii) > average perceived stress (4–5).

The level of sleep satisfaction was measured with the following question: “In the last week, how satisfactory was your sleep in terms of relieving your fatigue?”. The response options were very unsatisfactory (1), unsatisfactory (2), average (3), satisfactory (4), and very satisfactory (5). On the basis of the responses, participants were classified into the following two groups for multivariate logistic regression analyses: ≤ average satisfaction with sleep (1–3) and (ii) > average satisfaction with sleep (4–5).

### Statistical analysis

Sociodemographic characteristics, perceived stress, sleep satisfaction, and problematic Internet use were compared between the physically active and physically inactive groups using *χ*^2^-tests for categorical variables and independent *t*-tests for continuous variables. Adjusted odds ratios (AOR) and 95% confidence intervals (CI) were derived from a series of logistic regression analyses using problematic Internet use, sleep satisfaction, and level of stress as the main outcome variables and level of PA as the principal predictor after adjusting for age, sex, place of residence, perceived academic performance, perceived family economic status, parents’ level of education, and BMI. All variables were concurrently entered in the model. Then, the Sobel test [23] was used to explore the mediation of the relationship between PA and problematic Internet use by sleep satisfaction and stress. All statistical analyses were performed using SPSS (version 21.0; SPSS Inc., Chicago, IL), with statistical significance defined as an alpha level < 0.05.

## Results

Of 72,328 respondents, 64.5% met criteria for the physically inactive group and 35.5% met criteria for the physically active group. Among 25679 physically active subjects, 6591 (9.0% of all participants) met both criteria for being active, 17703 (24.2% of all participants) only met criteria for vigorous PA, and 1365 (1.9% of all participants) only met criteria for moderate PA. Table 1 shows the distribution of frequencies for moderate and vigorous PA.

**Table 1:** **Distribution of frequencies for moderate and vigorous physical activity (PA) in 73,238 Korean adolescents**

	Vigorous PA, N (%)	Moderate PA, N (%)
None	21726 (29.7)	20284 (27.7)
1 day per week	13804 (18.8)	16357 (22.3)
2 days per week	13414 (18.3)	14840 (20.3)
3 days per week	10932 (14.9)	10308 (14.1)
4 days per week	4045 (5.5)	3493 (4.8)
5 or more days per week	9317 (12.7)	7956 (10.9)

Table 2 presents the characteristics by group. Compared with physically inactive subjects, physically active participants were more likely to be male and younger and to have higher perceived academic performance, perceived family income, parental educational levels, and BMIs. Physically active subjects had significantly higher levels of sleep satisfaction, lower levels of stress, and lower scores on the KS scale than did inactive subjects. However, the effect sizes were very small (Cohen’s d: 0.15 for sleep satisfaction, 0.18 for perceived stress, and 0.05 for KS scale scores).

**Table 2:** **Characteristics according to physical activity status in 73,238 Korean adolescents**

	Inactive	Active		
	(N = 47549)	(N = 25689)	***X*** ^2^/t	p
Sex, N (%)			5179.97	<0.001
Male	20283 (42.7)	18108 (70.5)		
Female	27266 (57.3)	7581 (29.5)		
Residential area, N (%)			20.61	<0.001
Rural	5712 (12.0)	3344 (13.0)		
Small city	17125 (36.0)	8941 (34.8)		
Large city	24712 (52.0)	13404 (52.2)		
Age (years), Mean (SD)	15.31 (1.74)	14.60 (1.67)	53.68	<0.001
Perceived academic performance, N (%)			101.06	<0.001
Very high	5015 (10.5)	3344(13.0)		
High-middle-low	36705 (77.2)	19242(74.9)		
Very low	5829 (12.3)	3103(12.1)		
Socio-economic status, N (%)			704.97	<0.001
Very high	2448 (5.1)	2330 (9.1)		
High	9850 (20.7)	6444 (25.1)		
Middle	23044 (48.5)	11209 (43.6)		
Low	9179 (19.3)	4211 (16.4)		
Very low	3028 (6.4)	1495 (5.8)		
Father’s education level, N (%)			31.41	<0.001
Complete middle school or less	2918 (7.4)	1485 (7.1)		
Complete high school	17273 (44.0)	8727 (41.9)		
Complete college or more	19079 (48.6)	10622 (51.0)		
Mother’s education level, N (%)			129.43	<0.001
Complete middle school or less	2922 (7.4)	1390 (6.7)		
Complete high school	22755 (57.8)	11132 (53.8)		
Complete college or more	13686 (34.8)	8161(39.5)		
Body mass index, Mean (SD)	20.44 (2.93)	20.56 (3.08)	−5.23	<0.001
Sleep satisfaction (1–5)	2.78 (1.10)	2.95 (1.13)	−19.20	<0.001
Perceived stress (1–5)	3.45 (0.92)	3.28 (0.99)	22.96	<0.001
KSscale scores (20–80)	30.20 (9.40)	29.74 (9.69)	6.18	<0.001

KSscale, Internet Addiction Proneness Scale for Youth–Short Form.

Table 3 shows the results of logistic regression models treating sleep satisfaction, perceived stress, and problematic Internet use as the main outcome variables and level of PA as the principal predictor. Physically active subjects were more likely to express satisfaction with their sleep (*AOR* = 1.13; 95% *CI* = 1.08, 1.18), less likely to feel stress in their lives (*AOR* = 0.89; 95% *CI* = 0.86, 0.93), and less likely to be a problematic Internet user (*AOR* = 0.78; 95% *CI* = 0.73, 0.82) after controlling for age, sex, place of residence, perceived academic performance, perceived family economic status, parents’ level of education, and BMI.

**Table 3:** **Associations (AOR, 95% CI) of physical activity with perceived stress, sleep satisfaction, and problematic internet use**

	Inactive	Active			Active ***vs***. Inactive		
	N (%)	N (%)	***X*** ^2^	p	AOR	95% CI	p
Sleep satisfaction	12136 (25.5)	8191 (31.9)	336.67	<0.001	1.13	1.08, 1.18	<0.001
Perceived stress	22086 (46.4)	10008 (39.0)	380.13	<0.001	0.89	0.86, 0.93	<0.001
Problematic internet use	6346 (13.3)	3108 (12.1)	23.10	<0.001	0.78	0.73, 0.82	<0.001

AOR, odd ratio adjusted for age, sex, residing region, perceived academic performance, family economic status, parents’ level of education, and body mass index; CI, confidence interval.

**Figure 1:**
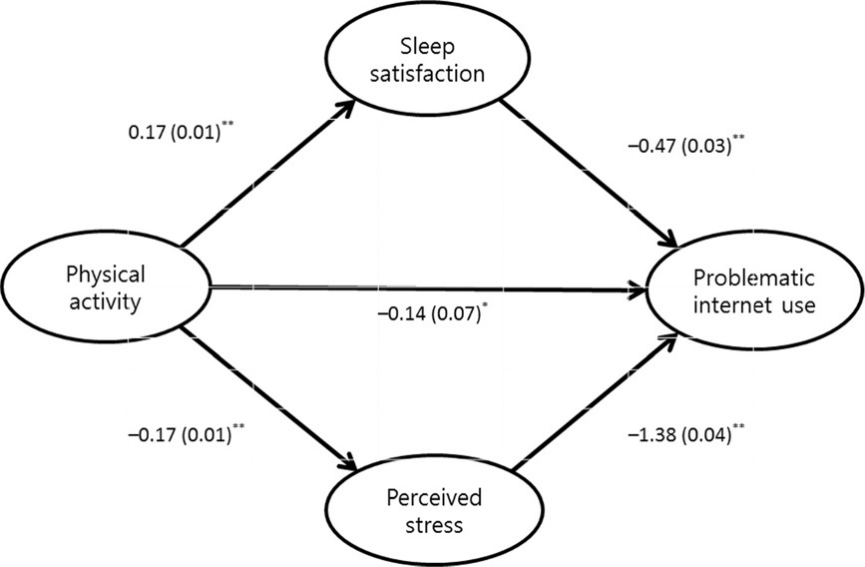
**Associations [** ***B*** **coefficients (** ***SE*** **)] between physical activity, perceived stress, sleep satisfaction, and problematic internet use.** Footnote: Sobel test was used (Z = −4.315, p < 0.001).^*^p < 0.05, ^**^p < 0.001.

The Sobel test showed that the inverse association between PA and problematic Internet use was partially mediated by increased sleep satisfaction or decreased perceived stress (*Z* = −4.315, *p* < 0.001). As shown in Figure 1, PA predicted higher levels of sleep satisfaction (unstandardized coefficients, *B* = 0.17; 95% *CI* = 0.15, 0.19; *P* < 0.001), which predicted lower KS scale scores (*B* = −0.47; 95% *CI* = −0.53, −0.39; *P <* 0.001). PA also predicted lower levels of perceived stress (*B* = −0.17; 95% *CI* = −0.19, −0.16; *P <* 0.001), which predicted lower KS scale scores (*B* = 1.38; 95% *CI* = 1.29, 1.45; *P <* 0.001). In addition to these indirect effects via increased sleep satisfaction and reduced perceived stress, PA directly predicted lower scores for problematic Internet use (*B* = −0.14, 95% *CI* = −0.28, −0.02; *P* = 0.049), but this direct effect was much less significant than was its indirect effect through perceived sleep and stress (*B* = −0.31, 95% *CI* = −0.34, −0.29; *P* = 0.003).

## Discussion

The main findings of this study were that PA was associated with higher levels of sleep satisfaction, lower levels of perceived stress, and a low risk of problematic Internet use. The inverse association between PA and problematic Internet use was partially mediated by sleep satisfaction and stress.

Previous studies of the effect of PA on mental health in children and adolescents have focused on depression, anxiety, and self-esteem [5]. Systematic reviews indicate that PA is associated with reduced depression and anxiety [3, 5, 24, 25] and higher self-esteem [4, 5, 26] in children and adolescents. Participation in PA has been also associated with positive mood and greater psychological well-being [2–4, 14]. Additionally, PA is positively associated with restorative sleep and ameliorating sleep disturbances [20]. The findings of the present study confirm these positive effects of PA on mental health. In addition to the favorable effects on sleep and stress that were demonstrated in previous studies, a protective effect of PA on problematic Internet use was identified in this study. However, the favorable effect of PA on problematic Internet use was primarily mediated by increased sleep satisfaction and reduced stress.

Two main hypotheses about the mechanism through which PA positively influences mood and reduces stress have been proposed. According to the first hypothesis, high-intensity exercise has been shown to result, at least in the short term, in increased cortical blood flow, endorphin release, and epinephrine and norepinephrine synthesis [17]. According to the second hypothesis, exercise allows for a discharge of hostility, reduces emotional strain, and promotes a sense of mastery and increased self-esteem [17, 27]. In terms of the possible biological mechanisms through which PA positively influences sleep, it has been suggested that strenuous exercise results in a significant increase in the proportion of slow-wave sleep, a reduction in stage-2 sleep, greater sleep efficiency, and shorter sleep-onset latency [28]. Other hypotheses hold that the sleep-promoting effects of PA are mediated by psychological functioning [29] or by reduced symptoms of depression and anxiety [6, 30, 31].

Despite the positive effect of PA and sleep on mental health, including their protective effects on problematic Internet use, many adolescents reduce their PA levels and sleep quantity during this developmental stage [32, 33]. This is particularly true in South Korea, where the academic stress experienced by middle- and high-school students is extremely high. The average amount of time that Korean adolescents aged 15–24 years devoted to studying was 7 hours 50 minutes per day, which was much longer than the average number of hours devoted to studying by adolescents in other countries, which ranged from 3 to 6 hours [34]. In contrast, Korean adolescents spent less time sleeping (7 hours 30 minutes per day) and exercising (13 minutes per day) than did adolescents in other countries, including England, America, Holland, Sweden, and Finland [34]. Considering these statistics and the results of the present study, it is suggested that low levels of PA, poor sleep, and high academic stress may contribute to the high prevalence of problematic Internet use in Korean adolescents.

The limitations of this study include a lack of reliability and validity testing for the single-item surveys on stress and sleep satisfaction. Moreover, information about the frequency and intensity of PA was provided gathered via self-reports rather than direct observations, which may have led to reporting bias. Additionally, because the data were cross-sectional, no inferences about causality are possible at this time. Several possible bidirectional or interactive relationships could link PA with sleep satisfaction, stress, and problematic Internet use. One possibility is that experience of stress [35] and poor quality sleep [36] may impair efforts to be physically active and extensive use of the Internet may replace time spent in PA [15]. Finally, there might be factors such as life-style factors and personality traits, not accounted for in this study, which co-vary with PA and are true pathways to stress and problematic Internet use. However, the strength of the present study is its inclusion of data from 75,643 adolescents from a nationally representative sample in South Korea. Thus, the relationship of PA with sleep, stress, and problematic Internet use can be generalized to all Korean adolescents.

## Conclusions

The results of the present study indicate a negative association between level of PA and risk of problematic Internet use via the mediation of sleep satisfaction and stress. PA may be helpful to improve sleep, relieve stress, and decrease problematic internet use in adolescents. Future prospective studies are needed to determine the causal relationship between PA and psychiatric conditions, including problematic Internet use, in adolescents.
